# Regulatory Approaches to Cybersecurity Risk Management for AI-Enabled Medical Device Software in Korea, the United States, and the European Union: Comparative Document Analysis

**DOI:** 10.2196/94846

**Published:** 2026-07-30

**Authors:** Saera Jung, Kihong Son

**Affiliations:** 1 Department of Artificial Intelligence, University of Science and Technology (UST), 217 Gajeong-ro, Yuseong District, Daejeon, Republic of Korea, 82 10-8633-1544; 2 Qualitics Consulting, Seoul, Republic of Korea; 3 Medical Information Research Section, Electronics and Telecommunications Research Institute (ETRI), Daejeon, Republic of Korea

**Keywords:** cybersecurity, medical device software, software as a medical device, artificial intelligence, generative AI, risk management, ISO 14971, IEC 81001, AAMI TIR57, regulatory science, post-market surveillance

## Abstract

**Background:**

Software-based and AI-enabled medical devices are increasingly networked and updatable, expanding the attack surface and making cybersecurity governance intersect with quality management and postmarket oversight. Regulated device risk management nevertheless remains primarily oriented toward patient-safety harms under ISO 14971 frameworks, which may not fully capture cybersecurity risks affecting data integrity, system resilience, or service continuity.

**Objective:**

This study aimed to compare how Korea’s Ministry of Food and Drug Safety (MFDS), the US Food and Drug Administration (FDA), and the European Union/Medical Device Coordination Group (EU/MDCG) define and operationalize cybersecurity for medical device software across premarket review and postmarket surveillance, and to identify informatics-relevant gaps between safety vigilance and vulnerability-focused cybersecurity practice.

**Methods:**

We conducted a qualitative comparative document analysis of 10 jurisdiction-specific regulatory and guidance documents (MFDS: n=2, FDA: n=4, and EU/MDCG: n=4), supplemented by cross-sectoral instruments and peer-reviewed literature. Using a common analytic framework informed by functional comparative legal analysis, we mapped (1) conceptual scope (definitions and life cycle boundaries), (2) premarket operationalization (required artifacts and evidence such as threat modeling, software bills of materials, and vulnerability management plans), and (3) postmarket operationalization (monitoring, reporting, and update governance).

**Results:**

Of the 10 documents analyzed (MFDS: n=2, FDA: n=4, and EU/MDCG: n=4), all 3 jurisdictions converged on protecting confidentiality, integrity, and availability of data and device functions but embedded these expectations in different regulatory architectures. MFDS emphasized documentation completeness aligned with ISO 14971 risk management; the FDA framed cybersecurity as quality-system and design-control activities spanning the total product life cycle, including statutory requirements for “cyber devices” under Federal Food, Drug, and Cosmetic Act section 524B; and the European Union treated cybersecurity as an extension of safety under the Medical Device Regulation (MDR) and In Vitro Diagnostic Regulation (IVDR), interpreted through MDCG guidance, with additional cross-sector obligations from the Network and Information Security 2 (NIS2) Directive and the General Data Protection Regulation (GDPR). A common limitation was that vigilance pathways were largely triggered by patient-harm thresholds, whereas vulnerabilities and near-miss security events were often managed through parallel information-security processes. Mapping to ISO 13485 Clauses 7.3 and 8 indicated that integration of cybersecurity controls into existing quality management system (QMS) processes is feasible but not consistently mandated.

**Conclusions:**

Across the 3 jurisdictions examined in this study, regulatory approaches to medical device cybersecurity show definitional alignment but operational fragmentation at the interface between patient-safety vigilance and vulnerability-centric cybersecurity practice. Within the limits of this document-based analysis, the findings suggest that integrating cybersecurity as an interoperable process within the QMS—linking vulnerability monitoring, incident response, and software update controls to corrective and preventive action (CAPA) and change control—and expanding postmarket surveillance to incorporate vulnerability and performance signals could support more trustworthy deployment of regulated AI-enabled medical software.

## Introduction

Software-based medical devices, including AI-enabled systems, are increasingly deployed as workflow-critical components in clinical environments. As these products become more connected, updateable, and integrated with hospital networks and cloud services, cybersecurity becomes a core determinant of reliability, availability, and clinical trust. This creates a practical need to understand how cybersecurity expectations are translated into evidence requirements, quality processes, and postmarket controls for regulated medical device software (MDSW).

In this paper, we use the term “AI-enabled medical device software” to refer to MDSW that incorporates one or more AI or machine learning components. This includes software as a medical device (SaMD) where the software itself is the regulated product, software in a medical device where AI is embedded in a hardware device, adaptive or continuously learning algorithms, large language and large multimodal models used for medical purposes, and clinical decision-support functions that meet the regulatory definition of a medical device. Where jurisdictional terminology differs (eg, MDSW under the European Union Medical Device Regulation [MDR] and In Vitro Diagnostic Regulation [IVDR]), the corresponding national term is used.

SaMD has advanced rapidly and is increasingly enabling high-performance medical imaging and clinical workflows. Analyses of US and Australian regulatory databases indicate that SaMD is widespread yet not readily distinguishable from other medical-device submissions, partly because regulatory vocabularies and adverse-event codes rely on coarse categories such as “computer software problem” [1,2]. This lack of resolution constrains informatics-enabled safety improvement, including the ability to trace failure modes to software architecture, evaluate downstream effects of cybersecurity weaknesses on clinical performance, and operationalize continuous monitoring approaches that depend on structured, comparable event data.

**Figure 1.**
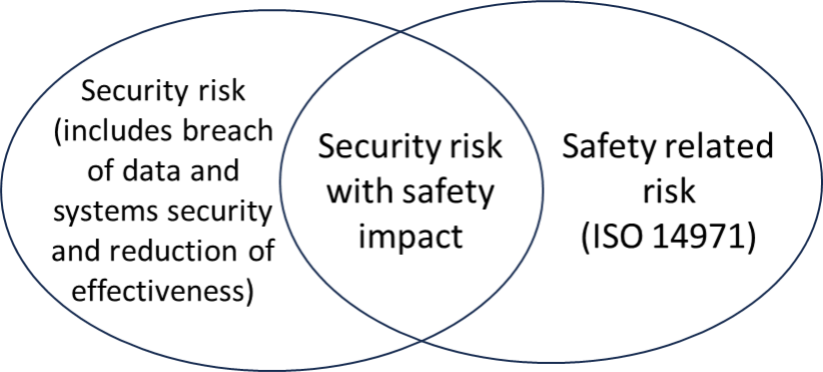
Conceptual relationship between security risk and safety-related risk in medical devices. This diagram, adapted from Association for the Advancement of Medical Instrumentation TIR57:2016, illustrates that only a subset of cybersecurity risks overlaps with safety-related risks as defined under ISO 14971. While security risks may affect confidentiality, integrity, availability, or system effectiveness, only those that result in patient harm are captured within traditional medical-device safety risk management frameworks.

In parallel, the broader health information technology environment has been repeatedly disrupted by ransomware and data-breach incidents. Thematic analyses of attacks on US hospitals and clinics demonstrate substantial operational and financial impact, even when direct patient harm is difficult to quantify [3,4]. Studies of connected medical devices and hospital networks have documented widespread exposure to known vulnerabilities, highlighting that cyber risk arises not only from individual devices but from their integration into complex sociotechnical systems [5-10] (Figure 1). These realities motivate a comparative examination of how regulators operationalize cybersecurity across premarket review and postmarket surveillance for AI-enabled MDSW.

Regulators and standard-setting bodies have responded by issuing guidance, standards, and frameworks for medical device cybersecurity. These include the National Institute of Standards and Technology (NIST) Cybersecurity Framework 2.0, International Electrotechnical Commission (IEC) 81001-5-1 [11] on health-software safety and security, Association for the Advancement of Medical Instrumentation (AAMI) TIR57 [12] on risk management for security in medical devices, the IEEE/UL standards for connected diabetes devices and clinical internet of things, and IEC 62443 and IEC/TR 60601-4-5 for security in industrial and medical control systems [11-16]. At the same time, cross-sector instruments such as the EU (European Union) Network and Information Security 2 (NIS2) Directive, the AI Act, the Cybersecurity Act, and the General Data Protection Regulation (GDPR) have established horizontal cybersecurity and data-protection requirements for products with digital elements [17-21].

Despite this proliferation of documents, a recent scoping review concluded that current regulations and standards offer only high-level statements on how cybersecurity should be reflected in benefit-risk analysis for medical devices, and rarely provide systematic methods for incorporating cyber risk into regulatory decision-making [22]. Other studies have questioned whether existing technical controls, such as those in ISO/IEC 80001-2-2, are sufficient for contemporary networked medical environments [23-26]. Work on SaMD postmarket surveillance and on clinical monitoring of AI systems likewise points to gaps between regulatory expectations and real-world safety monitoring [27-29].

Korea has recently published dedicated licensing guidelines for large language and multimodal models as medical devices [30-32], whereas equivalent generative AI–specific guidance in the United States and the European Union is still emerging. These contrasting trajectories underscore the need to reconcile traditional, safety-oriented risk management with broader cybersecurity governance for AI-enabled medical devices.

The aim of this study was to compare how 3 major regulatory systems—Korea’s Ministry of Food and Drug Safety (MFDS), the US Food and Drug Administration (FDA), and the EU/Medical Device Coordination Group (EU/MDCG)—define and operationalize cybersecurity for MDSW, including AI and generative AI components, across premarket review and postmarket surveillance. Building on this comparison, we sought to (1) characterize structural misalignments between ISO 14971 [33] device risk management and the broader cybersecurity risk space, (2) identify informatics-relevant artifacts (eg, threat modeling, software bills of materials, logging and telemetry, corrective and preventive action [CAPA] linkage, and change control) that can support interoperable life cycle governance, and (3) propose directions for aligning these regimes while preserving their distinct objectives.

## Methods

### Study Design

We performed a qualitative comparative document analysis to examine how cybersecurity is defined and operationalized for AI-enabled MDSW across Korea, the United States, and the European Union. The unit of analysis was an authoritative regulatory, guidance, or standards document that specifies expectations for cybersecurity-related risk management, evidence generation, and postmarket controls for regulated MDSW. The study design was informed by functional comparative legal research [34], in which legal instruments addressing the same regulatory function across jurisdictions are compared on the basis of the function they perform rather than their formal classification.

All 3 jurisdictions treat medical devices as a regulated industry that requires formal market authorization—premarket approval (PMA), clearance, or licensing—before commercial distribution. Each system pairs primary binding law (Korea: the Medical Device Act, its Enforcement Decree, and Enforcement Rules; United States: the Federal Food, Drug, and Cosmetic Act and FDA implementing regulations; and European Union: the MDR and IVDR) with implementing guidance documents (MFDS guidelines, FDA guidance documents, and MDCG guidance). Although these guidance documents are formally nonbinding in each system, in practice compliance with relevant guidance is required to obtain market authorization; FDA premarket review effectively expects conformity with applicable FDA guidance, MFDS approval review applies the relevant MFDS guidelines, and notified body assessment under MDR/IVDR consults MDCG guidance for interpretive expectations.

This shared regulatory architecture—binding primary law operationalized through compliance-required guidance, applied to the same regulated category of medical devices—provides the basis of functional equivalence on which our cross-jurisdictional comparison rests. The 10 documents in Table 1 were selected because each performs an equivalent regulatory function in its respective jurisdiction, irrespective of its formal classification.

These EU horizontal instruments (NIS2 Directive, AI Act, GDPR, and Cybersecurity Act) were not included in the 10-document core corpus listed in Table 1 but were analyzed as complementary contextual sources to characterize the regulatory environment within which MDR/IVDR Annex I §17.2 operates.

Cross-sectoral instruments (NIST Cybersecurity Framework 2.0, IEC 81001-5-1, AAMI TIR57, ISO 13485 [35], ISO 14971, ISO 27001, ISO 31000, ISO 42001, IEC 62443, and IEC/TR 60601-4-5) and peer-reviewed literature were treated as background and contextual material rather than part of the core corpus, and are cited where relevant in Results and Discussion sections.

**Table 1.** Core corpus of jurisdiction-specific regulatory and guidance documents analyzed (n=10).

Jurisdiction	Title	Issuing body	Year
Korea	Guideline for Cybersecurity in Medical Devices Premarket Submissions	MFDS^a^	2025
Korea	Guideline for Generative AI in Medical Devices Premarket Submissions	MFDS	2025
United States	Cybersecurity in Medical Devices: Quality System Considerations and Content of Premarket Submissions	FDA^b^	2026
United States	Postmarket Management of Cybersecurity in Medical Devices	FDA	2016
United States	Artificial Intelligence-Enabled Device Software Functions: Lifecycle Management and Marketing Submission Recommendations (draft guidance)	FDA	2025 (draft)
United States	Federal Food, Drug, and Cosmetic Act, Section 524B (“cyber devices” requirements)	US Congress / FDA	2023
European Union	Regulation (EU) 2017/745 on medical devices (MDR^c^), Annex I (general safety and performance requirements)	European Parliament & Council	2017
European Union	Regulation (EU) 2017/746 on in vitro diagnostic medical devices (IVDR^d^), Annex I	European Parliament & Council	2017
European Union	MDCG^e^ 2019‐16 Rev.1: Guidance on Cybersecurity for Medical Devices	MDCG	2020
European Union	MDCG 2025‐6: FAQ on the interplay between MDR, IVDR, and the AI Act	MDCG	2025

a MFDS: Ministry of Food and Drug Safety.

b FDA: US Food and Drug Administration.

c MDR: Medical Device Regulation.

d IVDR: In Vitro Diagnostic Regulation.

e MDCG: Medical Device Coordination Group.

### Data Sources and Selection

We assembled a jurisdiction-specific regulatory corpus from publicly available sources, including laws and implementing regulations, regulator-issued guidance, and regulator-endorsed standards frameworks relevant to medical device cybersecurity and software life cycle controls.

To contextualize regulatory expectations, the corpus was supplemented with peer-reviewed literature on (1) SaMD presence, recalls, and software-related adverse events in regulatory databases [1,2,36,37]; (2) vulnerabilities, attacks, and connectivity issues in hospitals and networked medical devices [5-10]; and (3) benefit-risk analysis and postmarket monitoring challenges for software and AI-enabled systems [22,27-29,36].

Inclusion criteria for the core regulatory corpus were as follows: (1) the document was issued by a competent regulatory authority (MFDS, FDA, or EU/MDCG) or was a standards document explicitly referenced by such an authority; (2) the document directly addressed cybersecurity, software life cycle, or AI governance for medical devices; (3) the document was the most recent finalized or draft version available as of March 7, 2026; and (4) an authoritative English version (or, for MFDS documents, the original Korean version with an authorized English translation reference) was publicly accessible. Exclusion criteria were (1) draft documents that had been formally withdrawn or superseded, (2) sector-specific guidance unrelated to MDSW (eg, automotive and energy), and (c) opinion pieces or commentaries without regulatory standing. The 10 documents that constitute the core corpus are listed in Table 1.

### Analytical Approach

To enable cross-jurisdiction comparison, we applied a common analytic framework that mapped document content into three domains: (1) conceptual scope (definitions, protected assets, threat assumptions, and life cycle coverage), (2) premarket operationalization (required artifacts and evidence such as secure design controls, threat modeling, software bills of materials, and vulnerability disclosure and management plans), and (3) postmarket operationalization (monitoring, incident and vulnerability handling, update governance, and mechanisms linking cybersecurity actions to quality management processes such as CAPA and change control). The mapping followed the principles of functional comparative legal analysis; rather than comparing legal terms in isolation, we compared the regulatory functions performed by each instrument across the medical device total product life cycle.

**Figure 2.**
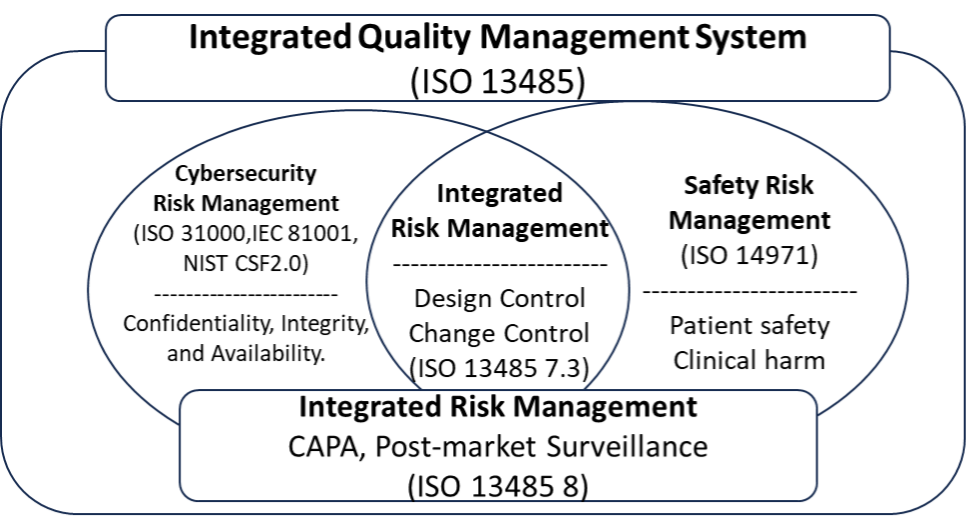
Conceptual model of an integrated quality management system (QMS) for medical devices. This figure illustrates how safety risk management based on ISO 14971 and cybersecurity risk management aligned with ISO 31000-based frameworks can be maintained as conceptually distinct systems while being operationally integrated within a single QMS. Shared QMS processes—such as design control, change management, corrective and preventive action, and postmarket surveillance—provide a unified governance structure across the total product life cycle, enabling consistent oversight of both patient safety and cybersecurity risks.

Furthermore, 2 analytic steps supported the synthesis presented in the Discussion section. First, we mapped the cybersecurity-relevant requirements identified in each jurisdiction onto the quality management system (QMS) clauses of ISO 13485:2016, with particular attention to Clause 7.3 (design and development) and Clause 8 (measurement, analysis, and improvement). This mapping is presented in Table 2. Second, we developed a conceptual model (illustrated in Figure 2) that depicts how ISO 14971–aligned safety risk management and ISO 31000-aligned cybersecurity risk management can be maintained as conceptually distinct processes while being operationally integrated within a single QMS.

Together, the predetermined change control plan (PCCP) and the Clause 7.3/Clause 8 axis convert cybersecurity risk management from a 1-time premarket exercise into a sustainable life cycle process that remains coherent across software updates, AI model retraining, and evolving threat landscapes.

**Table 2.** Summary mapping of safety and cybersecurity risk management within ISO 13485 (Clauses 7.3 and 8).

ISO 13485 clause	QMS^a^ process	Safety risk management (ISO 14971)	Cybersecurity risk management (ISO 31000-aligned)	Integrated QMS interpretation
7.3	Design and development	Identification and control of hazards affecting patient safety and clinical performance throughout design and development.	Identification of cybersecurity threats and vulnerabilities affecting confidentiality, integrity, availability, and system resilience during design.	Distinct safety and cybersecurity risks are addressed in parallel within a unified design-control framework, enabling coordinated risk identification, control, verification, and validation.
8	Measurement, analysis, and improvement	Monitoring of safety performance, adverse events, and residual risks through postmarket surveillance and CAPA^b^.	Monitoring of vulnerabilities, security incidents, and performance degradation through continuous surveillance and corrective actions.	Postmarket activities provide a shared governance mechanism for both safety and cybersecurity risks, extending oversight beyond patient harm to system-level and life cycle risks.

a QMS: quality management system.

b CAPA: corrective and preventive action.

### Researcher Characteristics and Reflexivity

The 2 authors bring complementary backgrounds: SJ (MS, microbiology) works in regulatory and quality consulting for medical technologies and has hands-on experience preparing medical device submissions in Korea, the United States, and the European Union; KS (PhD) is a senior researcher and associate professor in medical informatics, AI in health care. Neither author is employed by, or has consulted for, any of the regulatory agencies whose documents are analyzed. We acknowledge that SJ’s submission experience may have introduced a practitioner-oriented framing, while KS’s academic perspective emphasized conceptual and informatics-oriented aspects. The 2 authors reviewed each jurisdiction’s documents independently using the shared analytic framework and reconciled differences through discussion.

### Ethical Considerations

This study analyzed publicly available legal, regulatory, and standards documents and did not involve human participants or identifiable personal data; therefore, institutional ethics approval and informed consent were not required.

## Results

### Cybersecurity Concepts and Definitions

Across Korea (MFDS), the United States (FDA), and the European Union (EU/MDCG), cybersecurity for AI-enabled MDSW was consistently framed around maintaining the confidentiality, integrity, and availability of information and device functions across the product life cycle (Table 3). However, it was operationalized through different regulatory architectures, which shaped how threat assumptions, required artifacts, and postmarket signals were specified and governed.

**Table 3.** Cybersecurity-related terms and definitions in medical device regulations, guidance, and standards (Korea, United States, European Union, and international standards).

Source (issuing authority; jurisdiction)	Definition (quoted or adapted)
Guideline for Cybersecurity in Medical Devices Premarket Submissions (MFDS^a^, Republic of Korea)	Security/Cybersecurity—a state in which information and systems are protected from unauthorized activities (eg, access, use, disclosure, interference, modification, or destruction), such that risks to authentication, access control, integrity, confidentiality, data flow, timely response, and availability are maintained at an acceptable level throughout the device life cycle (adapted from IEC^b^ TR 60601-4-5:2021).
Cybersecurity in Medical Devices: Quality System Considerations and Content of Premarket Submissions (FDA^c^, United States)	Cybersecurity—the process of preventing unauthorized access, modification, misuse, denial of use, or other unauthorized use of information stored in, accessed by, or transferred from a medical device to an external recipient (adapted from IEC 27032:2012).
Postmarket Management of Cybersecurity in Medical Devices (FDA, United States)	Cybersecurity—the ability to protect or defend the use of cyberspace from cyberattacks (adapted from NIST SP 800‐39).
MDCG^d^ 2019‐16 Rev.1 (EU/MDCG, European Union)	Cybersecurity (in the EU medical device framework)—the protection of devices and information systems from unauthorized access, use, disclosure, disruption, modification, or destruction, in order to provide confidentiality, integrity, and availability; cybersecurity risks are evaluated as a subset of “risk” under the MDR^e^/IVDR^f^, defined as the combination of the probability of occurrence of harm and the severity of that harm. MDCG 2019‐16 Rev.1 explicitly applies this risk concept to security-related risks within the EU regulatory context.
ISO 24971 Annex F (ISO)	Security—a condition resulting from the establishment and maintenance of protective measures that ensure inviolability against hostile acts or influences, whether intentional or unintentional (see IEC Guide 120:2018). In AAMI^g^ TIR57:2016 and IEC 80001-1:2010, security is described as an operational state in which information assets are reasonably protected with respect to confidentiality, integrity, and availability.

a MFDS: Ministry of Food and Drug Safety.

b IEC: International Electrotechnical Commission.

c FDA: US Food and Drug Administration.

d MDCG: Medical Device Coordination Group

e MDR: Medical Device Regulation.

f IVDR: In Vitro Diagnostic Regulation.

g AAMI: Association for the Advancement of Medical Instrumentation.

In Korea, MFDS’s cybersecurity review guidance treats cybersecurity as a life cycle state that protects device information and functions against unauthorized actions, positioning cybersecurity as part of device safety and essential performance and documenting it within technical documentation under an ISO 14971 risk-management approach [38].

In the United States, the FDA final guidance Cybersecurity in Medical Devices: Quality System Considerations and Content of Premarket Submissions (issued February 3, 2026) positions cybersecurity as quality-system and design-control activities spanning the total product life cycle and incorporates recommendations related to Federal Food, Drug, and Cosmetic Act section 524B for “cyber devices” [39]. Postmarket expectations are further articulated in FDA’s Postmarket Management of Cybersecurity in Medical Devices (December 2016) and align with NIST terminology that defines cybersecurity as the ability to protect or defend the use of cyberspace from cyberattacks.

In the European Union, MDR/IVDR Annex I embeds cybersecurity-related general safety and performance requirements across both premarket and postmarket phases, and MDCG 2019‐16 Rev.1 provides interpretive guidance for manufacturers on meeting those requirements within an ISO 14971 risk-management lens [40-42].

At the level of binding horizontal legislation, several EU instruments operate in parallel to MDR/IVDR under the New Legislative Framework, as recognized by the Commission’s 2022 Blue Guide; to satisfy the cybersecurity-related General Safety and Performance Requirements (GSPRs) of MDR/IVDR, manufacturers therefore also bear compliance responsibilities under the NIS2 Directive (EU) 2022/2555, the AI Act (EU) 2024/1689, the Cybersecurity Act (EU) 2019/881, and the GDPR (EU) 2016/679. MDCG 2025‐6 (issued June 2025 as a finalized FAQ document) interprets the interplay between MDR, IVDR, and the AI Act [43]. Cybersecurity, however, differs from the other essential safety attributes addressed in Annex I in that it cannot be fully internalized within the manufacturer’s own design and risk-management process. IEC 80001‐1 frames risk management for IT-networks incorporating medical devices as involving multiple stakeholders—responsible organizations as well as manufacturers—rather than a single party assuming full responsibility for the network’s key properties. European Union Agency for Cybersecurity (ENISA), whose mandate was made permanent by the Cybersecurity Act, issues technical guidance and operates EU cybersecurity certification schemes that manufacturers and notified bodies may invoke as evidence of the “state of the art” required by Annex I §17.2. The security-of-processing obligations of the GDPR apply horizontally to health data handled by AI-enabled devices and conceptually mirror the security-by-design expectation embedded in the GSPR. Accordingly, to achieve full GSPR compliance at the design stage, manufacturers must recognize cybersecurity-specific requirements that fall outside the scope traditionally addressed by ISO 14971-based safety risk management.

### Premarket Control of Software and AI-Enabled Devices

#### Overview

Premarket expectations converged on demonstrable secure development practices, including traceable security requirements, risk assessment, and verification and validation evidence. Jurisdictions differed primarily in how these expectations were presented (documentation checklists vs quality-system controls vs general safety and performance requirements) and in the explicitness of life cycle artifacts such as threat modeling, software bills of materials, coordinated vulnerability disclosure, and update governance.

Across the 3 jurisdictions, premarket pathways for medical devices vary by their regulatory purpose. Approval and clearance pathways—which include 510(k) clearance, De Novo classification, and PMA in the United States; notified-body conformity assessment for higher-risk classes under MDR/IVDR in the European Union [44]; and MFDS licensing review in Korea—involve substantive premarket evaluation of safety and effectiveness, including cybersecurity considerations. Registration or notification pathways—which include Class I exemptions in the United States, self-declaration of conformity for Class I devices in the European Union, and notification for low-risk devices in Korea—serve principally administrative tracking and do not entail equivalent substantive premarket review. Not all medical devices, therefore, undergo third-party conformity assessment or substantive cybersecurity review before market entry. The cybersecurity expectations summarized below apply to those device classes for which the relevant jurisdiction mandates substantive premarket review under the approval or clearance pathway, rather than to devices that are subject only to administrative registration.

#### MFDS (Korea)

The MFDS premarket cybersecurity requirements are set out in the Guideline for Cybersecurity in Medical Devices Premarket Submissions. The checklist items summarized in Table 4 are largely derived from the International Medical Device Regulators Forum (IMDRF) document Principles and Guidelines for Medical Device Cybersecurity and from the standards IEC 62443-4-2:2019 and IEC TR 60601-4-5:2021. By referencing these documents, the guideline aims to capture the technical characteristics of currently marketed products.

**Table 4.** Premarket cybersecurity elements required by MFDS^a^, FDA^b^, and the European Union.

Competent authority or requirement document	Key elements of cybersecurity	AI or generative AI specific notes
MFDS (Republic of Korea) / Guideline for Cybersecurity in Medical Devices Premarket Submissions (November 2024)	Identification and authentication; usage control; system integrity; data confidentiality; timely event response; resource availability.	Guideline for Generative AI in Medical Devices Premarket Submissions (January 2025). Risk management is applied according to ISO 14971:2019. Examples of AI-specific hazards include: (1) performance hallucination, inconsistency, irrelevancy, lack of uncertainty indicator, limited explainability and interpretability; (2) data quality issues such as incorrect data, mishandling of outliers, incomplete data, subjective data, inconsistent data, domain shift, data drift, and fragmented data; (3) bias, including selection bias, confounding variables, nonnormality, proxy variables, and implicit bias; (4) user-related risks such as overconfidence; (5) adaptive system behavior; and (6) other risks such as lack of operator knowledge.
FDA (United States) / Cybersecurity in Medical Devices: Quality System Considerations and Content of Premarket Submissions (February 3, 2026)	Authentication; (B) authorization; (C) cryptography; (D) code, data, and execution integrity; (E) confidentiality; (F) event detection and logging; (G) resiliency and recovery; (H) firmware and software updates.	Artificial Intelligence-Enabled Device Software Functions: Lifecycle Management and Marketing Submission Recommendations (draft guidance, January 2025). AI components can present cybersecurity risks including data poisoning, model inversion, model stealing, model evasion, data leakage, overfitting, model bias, and performance drift.
European Commission (European Union) / MDCG^c^ 2019‐16 Rev.1, Guidance on Cybersecurity for Medical Devices (December 2019)	IT security: confidentiality of information at rest and in transit; integrity, ensuring information authenticity and accuracy (ie, nonrepudiation); availability of processes, devices, data, and connected systems.	MDCG 2025‐6 (June 2025). The MDR^d^, IVDR^e^, and AI Act emphasize the need for robust cybersecurity measures in both the premarket and postmarket stages of high-risk medical device AI (MDAI).

a MFDS: Ministry of Food and Drug Safety.

b FDA: US Food and Drug Administration.

c MDCG: Medical Device Coordination Group.

d MDR: Medical Device Regulation.

e IVDR: In Vitro Diagnostic Regulation.

In all 3 jurisdictions, premarket cybersecurity evaluation is anchored in ISO 14971–based risk management but implemented through distinct instruments.

The listed items represent the minimum set of cybersecurity requirements that applicants are expected to address in their approval submissions. Where a particular requirement cannot be applied due to the characteristics of the product, the applicant must provide supporting documentation—such as the risk-management file, user manual, or design documentation—justifying the nonapplicability.

For AI-based devices, the guideline introduces the concept of “AI security,” described as adding cybersecurity requirements or selecting appropriate requirements from the detailed checklist for additional verification. However, the guideline does not specify AI-specific cybersecurity elements beyond this general instruction. In January 2025, Korea published licensing review guidelines for generative AI–enabled medical devices [31]. For premarket submissions, the guideline instructs sponsors to follow the existing Guideline for Cybersecurity in Medical Devices Premarket Submissions, while also recommending that risk management be tailored to the technologies implemented in the device by explicitly identifying threats unique to generative AI.

ISO 14971–based processes are fundamentally oriented toward patient safety and the clinical performance of medical devices. When risk assessment is conducted solely within that framework, clearly identified cybersecurity threats may be deprioritized if they are not expected to result in patient injury or unacceptable degradation of device performance. As a consequence, AI- and generative AI–specific threats that primarily affect information security or system integrity may be filtered out during premarket evaluation and become visible, if at all, only in the postmarket phase. This limitation reflects that the broader universe of software security and cybersecurity risks extends beyond the risk space formally captured by ISO 14971 [32,45,46].

#### FDA (United States)

In the premarket context, the FDA expects manufacturers to analyze the configuration and vulnerabilities of medical devices, develop threat models that may affect safety, and verify the effectiveness of corresponding countermeasures. Key cybersecurity activities in this process follow the risk-management approach described in 21 CFR 820.30(g) [47] on design controls and in the guidance document Cybersecurity in Medical Devices: Quality System Considerations and Content of Premarket Submissions. These expectations are consistent with the IMDRF guidance Principles and Practices for Medical Device Cybersecurity, particularly the recommendations on cybersecurity risk management throughout the device life cycle.

For AI-enabled devices, the FDA guidance further recommends that sponsors develop an explicit threat model as part of the premarket cybersecurity risk-management process. This model should identify AI-specific technical vulnerabilities and associated hazard scenarios, including risks such as data poisoning, model inversion, model stealing, model evasion, data leakage, overfitting, model bias, and performance drift. By structuring these vulnerabilities into a device-specific threat model that is integrated with the quality system, manufacturers can more systematically select security controls, define appropriate test methods, and specify monitoring indicators. When such a device-specific threat model is embedded in the quality system, it operationalizes life cycle cybersecurity risk management and provides a structured basis for postmarket surveillance and for updating controls as new vulnerabilities are identified. Although the FDA’s 2025 draft guidance on AI-enabled device software functions provides explicit examples of AI-specific cybersecurity threats, it is at the time of writing a draft document and does not yet carry the binding effect of a final guidance, a point we revisit in the Limitations section.

#### The EU

Although the EU MDR and IVDR do not contain a dedicated chapter labeled “cybersecurity,” cybersecurity requirements can be inferred from the general safety and performance requirements and from related guidance. As discussed above, EU guidance interprets cybersecurity through the ISO 14971 risk-management framework, treating loss of confidentiality, integrity, or availability as a potential source of harm.

Accordingly, the primary objective of premarket cybersecurity evaluation in the EU is to demonstrate that the device can achieve its intended purpose while maintaining an acceptable level of risk to patients and public health. Security-by-design principles and IT security measures required under MDR/IVDR Annex I are therefore expected to be embedded in the manufacturer’s ISO 14971–based risk-management process.

MDCG 2019‐16 frames IT security, information security, and operational security as key domains of cybersecurity risk for medical devices. In addition to the IEC 80001 series on networked medical systems, it explicitly refers to the Information Security Management Systems requirements of ISO/IEC 27001 [48] as an Annex III reference standard. MDCG 2025‐6, interpreting the MDR, IVDR, and AI Act, further emphasizes that robust cybersecurity measures are required in both the premarket and postmarket phases of high-risk medical device AI. In our analysis, we treat the NIS2 Directive, the GDPR, and ENISA-issued guidance and threat reports as cross-sectoral instruments that EU manufacturers must reference in order to fulfill the GSPRs of MDR Annex I (notably Sections 17.2 and 17.4 on information security and minimum IT requirements). Specifically, NIS2 obligations apply where a medical device manufacturer or a health care delivery organization meets the criteria of an essential or important entity; GDPR obligations apply whenever personal health data are processed; and ENISA-issued guidance is referenced as nonbinding sectoral interpretation [18,20,21]. A key analytic finding, however, is that although these instruments are referenced in MDCG guidance, they are limited primarily to the broader risk definition (eg, personal data protection, network, and information system resilience) and are not structurally integrated with the cybersecurity definition or operational expectations within the medical device regulatory framework itself. As a consequence, the architectural separation creates a structure in which the requirements of NIS2, GDPR, and ENISA guidance are formally acknowledged but are not readily operationalized within manufacturers’ device-centric quality management systems—a structural limitation that we revisit in the Discussion section.

Taken together, these documents suggest that, as in other industries, medical-device manufacturers may adopt ISO/IEC 27001 [48] and ISO/IEC 42001 [49] as harmonized standards by integrating cybersecurity and AI-specific risk management into their QMS. This implies that certain aspects of cybersecurity and AI risk management can appropriately be organized and governed as dedicated (yet QMS-aligned) management systems, rather than being handled only within traditional safety engineering processes.

### Postmarket Surveillance and Cyber-Incident Reporting

Postmarket mechanisms showed the clearest signs of fragmentation (Table 5): patient-safety vigilance systems emphasize incidents that result in, or threaten, patient harm, whereas cybersecurity practice often centers on vulnerabilities, exploit intelligence, and near-miss signals that may not immediately manifest as harm. Across jurisdictions, this boundary shaped what was captured, where it was reported, and how quickly mitigation could be coordinated across manufacturers, health care delivery organizations, and national cybersecurity agencies.

**Table 5.** Postmarket mechanisms for medical device incident reporting and cybersecurity-related risk collection across MFDS^a^, FDA^b^, and the European Union.

Regulatory authority	Definition of reportable medical device incidents Cybersecurity incident reporting (mandatory)	Cybersecurity incident reporting
MFDS (Republic of Korea)	Public notice of Serious Adverse Event: (a) causes death or a life-threatening condition, (b) requires hospitalization or prolongation of an existing hospital stay, (c) results in persistent or significant disability or incapacity, and (d) causes a congenital anomaly or birth defect.	Electronic intrusion events: mandatory reporting by manufacturers to MFDS Commissioner, health care service providers, and end users (MFDS Notice No. 2025-30 [50]); voluntary reporting by health care service providers to MFDS Commissioner and manufacturers; technical security activities documentation required
FDA (United States)	Medical Device Reporting (21 CFR Part 803.3(w)): a serious injury is an injury or illness that (1) is life-threatening, (2) results in permanent impairment of a body function or permanent damage to a body structure, or (3) necessitates medical or surgical intervention to preclude permanent impairment of a body function or permanent damage to a body structure. Permanent means irreversible impairment or damage to a body structure or function, excluding trivial impairment or damage.	Voluntary. There is no cybersecurity-specific reporting mandate, and cybersecurity events are captured only through the patient-harm filter of Medical Device Reporting (21 CFR Part 803) and reports of corrections and removals (21 CFR Part 806). Supplementary voluntary or indirect mechanisms include the Recent Medical Device Recalls database and safety alerts, the Voluntary Malfunction Summary Reporting (VMSR) program (final guidance, 2024), Quality Management System Regulation (QMSR) inspections and change-control reviews, and Cybersecurity and Infrastructure Security Agency (CISA) medical device cybersecurity alerts and advisories
European Member States (EU)	Regulation (EU) 2017/745 and Regulation (EU) 2017/746, Article 2: “serious incident” means any incident that directly or indirectly led, might have led, or might lead to (a) the death of a patient, user, or other person; (b) the temporary or permanent serious deterioration of a patient’s, user’s, or other person’s state of health; or (c) a serious public health threat.	Voluntary at the device level. Mandatory cybersecurity-incident reporting exists only at the system or operational level under the NIS2 Directive, not as device-specific vigilance, and device-level cybersecurity is captured only when an event becomes a serious incident under the MDR or IVDR. Supplementary mechanisms include good manufacturing practice change control and notified-body surveillance audits, the manufacturer's postmarket surveillance system (including Article 88 trend reporting), and European Union Agency for Cybersecurity (ENISA) sector-specific threat and incident reports.

a MFDS: Ministry of Food and Drug Safety.

b FDA: US Food and Drug Administration.

In Korea, the MFDS issued guidance on cybersecurity incident response for digital medical devices in April 2025 (MFDS Notice No. 2025-30) [50], which requires manufacturers to document technical security activities, including file validation, data security, cryptographic key management, security monitoring, and AI-specific security measures, and to prepare a software bill of materials and vulnerability management plan. Under the Digital Medical Products Act Article 13(2), electronic intrusion is broadly defined as any act affecting the safety, efficacy, or performance of digital medical devices through methods such as hacking, computer viruses, logic or mail bombs, denial-of-service attacks, or high-power electromagnetic interference; such events are subject to mandatory reporting to the MFDS Commissioner, health care service providers, and end users. However, the breadth of these obligations is anticipated to create a gap between policy intent and operational implementation in practice. Cybersecurity incidents that do not originate from the medical device itself are subject to separate reporting obligations to the Korea Internet & Security Agency (KISA). Furthermore, the guidance does not articulate an integrated governance mechanism linking these two distinct risk domains, cybersecurity risk management and patient-safety risk management, for postmarket products, a structural gap shared across all 3 jurisdictions examined in this study [50].

In the United States, device-related incidents are reported through the Medical Device Reporting system and corrections-and-removals provisions; data populate databases such as the Manufacturer and User Facility Device Experience database and recall listings. Because there is no cybersecurity-specific reporting mandate, cybersecurity incidents enter these mandatory channels only when they manifest as patient harm; otherwise, they surface through voluntary or indirect routes such as safety communications and recalls coordinated with the Cybersecurity and Infrastructure Security Agency (CISA).. Studies of digital-device product summaries and security features show increasing attention to cybersecurity, but also highlight variability in disclosure practices [36].

In the European Union, serious incidents and field safety corrective actions are reported under the MDR or IVDR, whereas cybersecurity incidents as such fall outside device vigilance and are addressed at the system level under NIS2, national computer security incident response teams (CSIRTs), and ENISA-led coordination; that is, they are not treated as device-specific cybersecurity reporting.National technical rules, such as Germany’s BSI TR-03161 for health care applications, add further layers of requirements [17,51,52]. The GDPR introduces substantial financial penalties for data breaches, indirectly shaping incentives for cybersecurity investment [21].

Across the 3 regions, these arrangements lead to fragmented information flows: device regulators mainly see harm-oriented incident reports, while cybersecurity agencies handle broader vulnerability and breach data. AI-specific clinical monitoring schemes are still in early stages and vary widely between jurisdictions.

## Discussion

### Principal Results

This comparative document analysis of 10 jurisdiction-specific regulatory and guidance instruments produced 3 main findings. First, the 3 jurisdictions show clear definitional alignment around protecting confidentiality, integrity, and availability of medical device information and functions. Second, despite this conceptual convergence, cybersecurity is operationalized predominantly through ISO 14971–based safety risk management, which prioritizes patient harm and device performance and therefore tends to filter out cyber risks whose primary impact is on data, services, or system-level resilience. Third, postmarket surveillance shows the greatest fragmentation: device vigilance and information-security reporting flow through different institutions, with limited integration of vulnerability and near-miss security signals into device regulators’ visibility. Together, these findings indicate that current regimes converge on what cybersecurity is for, but diverge on how cybersecurity is governed and surveilled across the life cycle.

### Misalignment Between ISO 14971–Oriented Safety Risk Management and Cybersecurity Risk Management

First, ISO 14971 primarily targets hazards that can be directly linked to patient injury or unacceptable degradation of device performance. As a result, cybersecurity threats that predominantly affect confidentiality, data integrity, or service availability—such as ransomware attacks on Picture Archiving and Communication Systems, exploitation of third-party communication stacks, or large-scale data exfiltration—may not be captured as device hazards unless a clear causal link to clinical harm is established [3-8,10].

On June 27, 2019, the FDA and a manufacturer jointly disclosed wireless radio-frequency communication vulnerabilities affecting the Medtronic MiniMed 508 and Paradigm insulin pumps, issuing an urgent safety notice and recommending replacement of older devices. Although no confirmed adverse patient events had occurred, the vulnerabilities could have enabled unauthorized modification of pump settings or insulin delivery via nearby wireless signals, prompting proactive mitigation [53,54]. Similarly, on October 1, 2019, the FDA announced the URGENT/11 (IPnet) communication-stack vulnerabilities, which affected not only individual medical devices but also hospital networks, creating risks of remote control, denial of service, and unauthorized information disclosure [54]. In both cases, regulatory intervention was triggered by the potential for systemic impact rather than by documented patient harm, underscoring that cybersecurity risks can materially affect clinical safety even before traditional ISO 14971 safety end points are breached.

Beyond these traditional cybersecurity threats, AI-enabled MDSW introduces additional risk vectors including training-data poisoning, model manipulation, adversarial inputs, and prompt-based exploitation, while social engineering remains among the most prevalent threats to medical device security and patient safety.

Second, many cybersecurity obligations relevant to medical devices arise outside the conventional device-regulation domain. Legislative and regulatory instruments, such as the NIS2 Directive, the AI Act, the Cybersecurity Act, the GDPR, and national technical rules, impose requirements on manufacturers, operators, and service providers irrespective of medical-device classification [17-21,52]. These frameworks implement different risk concepts—such as essential-service continuity, data protection, and systemic resilience—and distinct reporting triggers, including “significant incidents” or personal-data breaches, which do not map neatly onto ISO 14971 hazard categories or medical-device vigilance definitions. Consequently, cybersecurity events with substantial operational or societal impact may be managed through parallel information-security channels without being recognized as device-related risks.

Third, AI-enabled and generative AI systems introduce additional forms of risk that further strain existing categories. Threats, such as data poisoning, model inversion, hallucination, bias, and performance drift, can compromise both cybersecurity and clinical performance, yet current guidance often distributes these concerns across safety, performance, and data-protection sections without an integrated view of AI-specific cyber risk or explicit expectations for continuous monitoring [32,37,45]. As software-based medical devices evolve after deployment—shaped by user behavior, organizational policies, configuration changes, and environmental conditions—new vulnerabilities may emerge that are not captured by static performance metrics or adverse-event reports.

Taken together, these factors suggest that monitoring device performance and patient-harm outcomes alone may be insufficient for cybersecurity risk management. Postmarket surveillance may benefit from extending beyond the individual device to encompass the broader technical and organizational environment, including third-party software components, communication interfaces, configuration management, and abnormal data flows. Without such an expanded perspective, detection of emerging risks may be delayed, accountability blurred, and the overall benefit-risk profile of digital medical products—particularly in large-scale, multivendor health care environments—more difficult to assess.

### Governance Fragmentation and a Tiered, Mammography Quality Standards Act–Analogous Response

Across the 3 jurisdictions, device regulators, manufacturers, health care delivery organizations (HDOs), and national cybersecurity agencies—KISA, CISA, ENISA, and national CSIRTs—operate within distinct reporting structures: device regulators primarily receive harm-oriented incident reports, whereas cybersecurity agencies handle vulnerability disclosures and breach data that need not satisfy patient-harm thresholds. Within manufacturers, regulatory affairs, quality, product security, and AI engineering teams likewise sit under separate management systems, and within HDOs, information-security operations and clinical risk management rarely share a common escalation pathway. This bifurcation slows correlation between vulnerability disclosures and clinical impact, complicates coordinated mitigation when a single vulnerability affects multiple manufacturers or shared hospital infrastructure, and limits systematic learning from near-miss events that are visible only within the cybersecurity domain. Frameworks such as the NIST Cybersecurity Framework 2.0 articulate multistakeholder risk governance in principle but stop short of specifying the institutional mechanisms by which such coordination occurs in clinical practice. At the policy level, the European Commission’s 2025 action plan on the cybersecurity of hospitals and health care providers (COM/2025/10 final) represents one recent attempt to bridge these silos through coordinated information-sharing, incident-response support, and threat-intelligence services for the health care sector [55].

To operationalize this coordination, we propose an interlocking 3-party model that distributes responsibility across the actors already present in the regulatory ecosystem. Manufacturers would extend the PCCP [56] beyond algorithm performance characteristics to incorporate manufacturer-defined threat models, making cybersecurity assumptions and the envelope of acceptable change explicit and auditable. HDOs would route cybersecurity events to the relevant national cybersecurity agency while concurrently flagging—jointly with the manufacturer—any observed conditions that fall outside the manufacturer’s PCCP. Within HDOs, this dual reporting is most effectively executed through standing multidisciplinary AI-cybersecurity review committees that combine clinical, biomedical engineering, information security, and quality and risk-management expertise; such committees provide the institutional substrate for triaging cybersecurity events with potential safety implications, deciding whether to escalate, and maintaining auditable links between security advisories and clinical change management. This dual-channel, voluntary reporting closes the gap between patient-safety vigilance and vulnerability-centric surveillance, and provides regulators with structured signals at the intersection of clinical use and cyber exposure.

A useful institutional precedent for this facility-anchored, multidisciplinary review function is the United States Mammography Quality Standards Act (MQSA) of 1992 (Public Law 102‐539; 21 CFR Part 900) [57]. MQSA itself regulates mammography image quality and does not address AI-enabled medical devices or cybersecurity; the analogy invoked here is institutional rather than substantive. Specifically, §900.3(b)(1) requires accreditation bodies to meet reliability and objectivity criteria when evaluating mammography facilities, demonstrating that an independent, multidisciplinary expert review function can be situated at the facility level and maintained over more than 3 decades through codified accreditation standards. The proposed AI-cybersecurity review committees draw on this model in structural form only—independent, multidisciplinary, and facility-anchored—while their remit (cybersecurity exposure, AI-specific risk such as data poisoning and model drift, and PCCP envelope monitoring) is categorically distinct from mammography image quality. Adopting this architecture would add a coordinating institutional layer between manufacturer postmarket obligations and device-specific vigilance pathways, through which voluntary reports of incidents and PCCP deviations can be triaged by cybersecurity experts working alongside clinical reviewers. Over time, this approach would accumulate a body of real-world response cases that translates principle-level coordination into concrete operational practice, addressing the fragmented oversight identified across Korea, the United States, and the European Union without requiring harmonization of underlying device-regulation frameworks.

### Implications for Manufacturers: an Integrated Multistandard QMS

The facility-anchored architecture described above must be matched by a corresponding integration at the manufacturer’s management-system level. We propose that ISO 14971–based safety risk management be combined with ISO/IEC 27001 (information security) [48], ISO/IEC 42001 (AI management systems) [49], and ISO 31000 (enterprise risk management for assets, personal data, and service continuity) [58] within a single, harmonized medical device QMS. The feasibility of such multistandard integration is already established in regulatory practice: CEN/TR 17223 clarifies the relationship between EN ISO 13485 and the MDR/IVDR, and IMDRF guidance on risk-management integration provides a structured basis for aligning safety, security, and AI governance under common QMS controls. A cross-sector precedent is offered by the automotive industry, where ISO 9001/IATF 16949 functions as a quality backbone through which ISO/IEC 27001 and ISO/IEC 42001 are operationalized as distinct yet interoperable risk domains, with controls executed via shared QMS mechanisms (design inputs, supplier qualification, configuration and change management, internal audit, CAPA, and management review).

This integration materially broadens the universe of formally managed risk beyond patient harm. Cybersecurity threats that primarily affect confidentiality, data integrity, or service availability—including those framed through information-security or AI-governance lenses such as personal-data exposure, asset compromise, model integrity loss, and disruption of dependent clinical services—are explicitly recognized as managed risks within the QMS, rather than being deferred to parallel information-security processes outside the device file. The manufacturer’s risk register therefore captures both ISO 14971-relevant safety hazards and ISO 31000-relevant operational and informational risks, with bidirectional traceability where a cybersecurity event has potential safety consequences. This dual recognition addresses the structural limitation identified in our analysis, in which clearly characterized cybersecurity threats may be deprioritized when they do not breach traditional patient-harm thresholds.

Operationally, manufacturers can anchor this integrated approach in 2 complementary mechanisms. First, the PCCP—extended, as proposed above, to incorporate manufacturer-defined threat models—serves as the controlled instrument through which cybersecurity assumptions, acceptable change envelopes, and trigger conditions for re-evaluation are pre-specified for both algorithmic and security parameters. Second, the ISO 13485 design and life cycle clauses provide the sustaining backbone: Clause 7.3 (design and development) controls hazard and threat identification, security and AI-governance design inputs, and verification and validation of risk-control measures during development, while Clause 8 (measurement, analysis, and improvement) governs continuing monitoring of vulnerabilities, performance drift, and CAPA-linked improvement during the postmarket phase.

### Limitations

This study has several limitations. First, the analysis relies on publicly available regulatory documents and selected academic literature; we did not conduct stakeholder interviews or collect quantitative data on incident frequency or device outcomes. We partially mitigated this by triangulating regulatory documents with peer-reviewed analyses of recalls, vulnerabilities, and postmarket monitoring practices.

Second, the regulatory landscape is evolving rapidly, and several documents in our corpus—particularly the FDA’s 2025 draft guidance on AI-enabled device software functions—are likely to be revised or finalized after the analysis cutoff. We anchored inclusion to the most recent version available as of March 2026 and explicitly identified draft versus final status in Tables 1 and 4; findings may need to be revisited as new guidance is issued, especially for generative AI.

Third, much of the analysis relies on guidance documents that are generally nonbinding and reflect interpretive recommendations rather than directly enforceable obligations, with practical influence varying across jurisdictions. We have therefore framed these documents as expected interpretations of binding regulations rather than as enforcement standards in themselves.

Fourth, the functional comparative approach enables cross-jurisdictional comparison on shared regulatory functions but abstracts from the broader legal-cultural context of each instrument; our claims are accordingly confined to convergence and divergence in operational expectations rather than to underlying legal-systemic differences.

### Comparison With Prior Work

Our findings align with prior reviews that note limited operational integration of cybersecurity considerations into traditional benefit-risk and vigilance frameworks for medical devices [22,27,29,36].

### Recommendations and Future Work

On the basis of this comparative analysis, future research and policy work could usefully address: (1) empirical evaluation of whether integrating cybersecurity controls into ISO 13485-aligned QMS processes improves incident response timeliness, (2) development of shared reporting taxonomies that allow vulnerability and near-miss data to be exchanged between device regulators and national cybersecurity agencies without overburdening manufacturers, (3) jurisdiction-specific operational guidance on AI-cybersecurity threats (data poisoning, prompt injection, model inversion) for high-risk medical device AI, and (4) qualitative studies of how multidisciplinary hospital governance structures handle cybersecurity events with potential clinical impact.

### Conclusions

In this comparative document analysis of regulatory and guidance instruments from Korea, the United States, and the European Union, the three jurisdictions shared a common conceptual foundation for medical device cybersecurity centered on protecting confidentiality, integrity, and availability of information and system functions across the product life cycle, but operationalized this foundation through different regulatory architectures. When cybersecurity is implemented primarily through safety-oriented, ISO 14971-based regulatory frameworks, important categories of cybersecurity risk—particularly those affecting service continuity, data protection, and system-level resilience—may remain partially outside the scope of device-centric risk management.

Across the documents analyzed, cybersecurity oversight was distributed across multiple regulatory and institutional domains, including medical device regulation, information security, health information technology governance, and horizontal cybersecurity law. As a result, cybersecurity incidents and vulnerabilities that did not immediately manifest as patient harm tended to be managed through parallel reporting and surveillance mechanisms rather than through medical device vigilance systems. Within the limits of a document-based design, these findings indicate that aligning cybersecurity risk management with QMS processes—while maintaining conceptual distinction from safety risk management—offers a coherent direction for life cycle oversight of AI-enabled MDSW, and warrants empirical evaluation in subsequent studies.

## Supplementary material

10.2196/94846 Checklist 1 SRQR checklist.

## Abbreviations

AAMI: Association for the Advancement of Medical Instrumentation
CAPA: corrective and preventive action
CISA: Cybersecurity and Infrastructure Security Agency
CSIRT: computer security incident response team
ENISA: European Union Agency for Cybersecurity
EU: European Union
FDA: US Food and Drug Administration
GDPR: General Data Protection Regulation
GSPR: General Safety and Performance Requirements (EU MDR/IVDR Annex I)
HDO: health care delivery organization
IEC: International Electrotechnical Commission
IMDRF: International Medical Device Regulators Forum
IVDR: In Vitro Diagnostic Regulation
KISA: Korea Internet & Security Agency
MDCG: Medical Device Coordination Group
MDR: Medical Device Regulation
MDSW: medical device software
MFDS: Ministry of Food and Drug Safety
MQSA: Mammography Quality Standards Act
NIS2: Network and Information Security 2 Directive (Directive (EU) 2022/2555)
NIST: National Institute of Standards and Technology
PCCP: predetermined change control plan
PMA: premarket approval
QMS: quality management system
SaMD: software as a medical device

## References

[1] Ceross A, Bergmann J (2021). Tracking the presence of software as a medical device in US food and drug administration databases: retrospective data analysis. JMIR Biomed Eng.

[2] Ceross A, Bergmann J (2021). Evaluating the presence of software-as-a-medical-device in the Australian therapeutic goods register. Prosthesis.

[3] Munoz Cornejo G, Lee J, Russell BA (2024). A thematic analysis of ransomware incidents among United States hospitals, 2016–2022. Health Technol.

[4] Neprash HT, McGlave CC, Cross DA (2022). Trends in ransomware attacks on US Hospitals, clinics, and other health care delivery organizations, 2016-2021. JAMA Health Forum.

[5] Williams PA, Woodward AJ (2015). Cybersecurity vulnerabilities in medical devices: a complex environment and multifaceted problem. Med Devices (Auckl).

[6] Yaqoob T, Abbas H, Atiquzzaman M (2019). Security vulnerabilities, attacks, countermeasures, and regulations of networked medical devices—a review. IEEE Commun Surv Tutorials.

[7] Mejía-Granda CM, Fernández-Alemán JL, Carrillo-de-Gea JM, García-Berná JA (2024). Security vulnerabilities in healthcare: an analysis of medical devices and software. Med Biol Eng Comput.

[8] Bracciale L, Loreti P, Bianchi G (2023). Cybersecurity vulnerability analysis of medical devices purchased by national health services. Sci Rep.

[9] Willing M, Dresen C, Haverkamp U, Schinzel S (2020). Analyzing medical device connectivity and its effect on cyber security in German hospitals. BMC Med Inform Decis Mak.

[10] Ostermann M, Freyer O, Weinhold C, Martius K, Gilbert S (2025). How secure are your health devices-stopping wearables becoming a personal and national security risk?. NPJ Digit Med.

[11] (2021). IEC 81001-5-1:2021. health software and health IT systems safety, effectiveness and security—part 5-1: security—activities in the product life cycle. https://www.iso.org/standard/76097.html.

[12] (2023). Principles for medical device security—risk management. https://array.aami.org/doi/10.2345/9781570206122.

[13] (2024). The NIST Cybersecurity Framework (CSF) 2.0.

[14] IEEE/UL (2022). Standard for Wireless Diabetes Device Security—Information Security Requirements for Connected Diabetes Solutions.

[15] International Electrotechnical Commission (2021). Medical electrical equipment—part 4-5: guidance and interpretation—safety-related technical security specifications. https://cdn.standards.iteh.ai/samples/103054/238d13c1fb034cf7b9e2d765f95dc568/IEC-TR-60601-4-5-2021.pdf.

[16] (2023). IEEE/UL Standard for Clinical Internet of Things (IoT) Data and Device Interoperability with TIPPSS--Trust, Identity, Privacy, Protection, Safety, and Security. https://standards.ieee.org/ieee/2933/7592/.

[17] European Parliament, Council of the European Union (2024). Regulation (EU) 2024/2847 on horizontal cybersecurity requirements for products with digital elements (cyber resilience act). European Union.

[18] European Parliament, Council of the European Union (2022). Directive (EU) 2022/2555 on measures for a high common level of cybersecurity across the union (NIS 2 directive). European Union.

[19] European Parliament, Council of the European Union (2024). Regulation (EU) 2024/1689 laying down harmonised rules on artificial intelligence (artificial intelligence act). European Union.

[20] European Parliament, Council of the European Union (2019). Regulation (EU) 2019/881 on ENISA (the european union agency for cybersecurity) and on information and communications technology cybersecurity certification (cybersecurity act). Off J Eur Union.

[21] European Parliament, Council of the European Union (2016). Regulation (EU) 2016/679 on the protection of natural persons with regard to the processing of personal data and on the free movement of such data (general data protection regulation). Off J Eur Union.

[22] Freyer O, Jahed F, Ostermann M, Rosenzweig C, Werner P, Gilbert S (2024). Consideration of cybersecurity risks in the benefit-risk analysis of medical devices: scoping review. J Med Internet Res.

[23] Anderson S, Williams T (2018). Cybersecurity and medical devices: Are the ISO/IEC 80001-2-2 technical controls up to the challenge?. Comput Stand Interfaces.

[24] Granlund T, Vedenpaa J, Stirbu V, Mikkonen T On medical device cybersecurity compliance in EU.

[25] Androutsos C, Taylor S, Bernsmed K, Praça I, Bernardi S, Inácio PRM (2025). Communications in Computer and Information Science.

[26] Taylor S, Gilje Jaatun M, Bernsmed K A way forward for the MDCG 2019-16 medical device security guidance. https://dl.acm.org/doi/proceedings/10.1145/3652037.

[27] Feng J, Xia F, Singh K, Pirracchio R (2025). Not all clinical AI monitoring systems are created equal: review and recommendations. NEJM AI.

[28] Zinchenko VV, Arzamasov KM, Chetverikov SF (2022). Methodology for conducting post-marketing surveillance of software as a medical device based on artificial intelligence technologies. Sovrem Tekhnologii Med.

[29] Warraich HJ, Tazbaz T, Califf RM (2025). FDA Perspective on the regulation of artificial intelligence in health care and biomedicine. JAMA.

[30] Park SH, Dean G, Ortiz EM, Choi JI (2025). Overview of South Korean guidelines for approval of large language or multimodal models as medical devices: key features and areas for improvement. Korean J Radiol.

[31] (2025). Guidelines for approval and review of generative AI medical devices [Webpage in Korean]. Ministry of Food and Drug Safety (MFDS).

[32] Park SH, Kim N (2024). Challenges and proposed additional considerations for medical device approval of large language models beyond conventional AI. Radiology.

[33] (2019). Medical Devices — Application of Risk Management to Medical Devices.

[34] Michaels R, Reimann M, Zimmermann R (2006). The Oxford Handbook of Comparative Law.

[35] (2016). Medical Devices — Quality Management Systems — Requirements for Regulatory Purposes.

[36] Stern AD, Gordon WJ, Landman AB, Kramer DB (2019). Cybersecurity features of digital medical devices: an analysis of FDA product summaries. BMJ Open.

[37] Chen WP, Teng WG, Kuo CB (2025). Regulatory insights from 27 years of artificial intelligence/machine learning-enabled medical device recalls in the United States: implications for future governance. JMIR Med Inform.

[38] (2024). Guideline for cybersecurity in medical devices premarket submissions. Ministry of Food and Drug Safety (MFDS) [Webpage in Korean].

[39] (2026). Cybersecurity in medical devices: quality system considerations and content of premarket submissions. US Food and Drug Administration.

[40] European Parliament, Council of the European Union (2017). Regulation (EU) 2017/745 on medical devices (medical device regulation). Official Journal of the European Union.

[41] European Parliament, Council of the European Union (2017). Regulation (EU) 2017/746 on in vitro diagnostic medical devices (in vitro diagnostic regulation). Official Journal of the European Union.

[42] (2020). MDCG 2019-16 rev1: guidance on cybersecurity for medical devices. Medical Device Coordination Group Document.

[43] (2025). MDCG 2025-6: FAQ on the interplay between the medical devices regulation (MDR), the in vitro diagnostic medical devices regulation (IVDR), and the AI act. Medical Device Coordination Group.

[44] (2023). Industrie-Gemeinschaft Niederbayern [Archived]. Questionnaire “cybersecurity for medical devices—audit.

[45] Finlayson SG, Bowers JD, Ito J, Zittrain JL, Beam AL, Kohane IS (2019). Adversarial attacks on medical machine learning. Science.

[46] (2021). Application of risk management for IT-networks incorporating medical devices — part 1: safety, effectiveness and security in the implementation and use of connected medical devices or connected health software. https://cdn.standards.iteh.ai/samples/23742/d4db769296ac4179ba018ddbaf7c6b93/IEC-80001-1-2021.pdf.

[47] US Code of Federal Regulations Title 21, Chapter I, Subchapter H, Part 820 — Quality System Regulation, §82030(g) Design Validation. https://www.ecfr.gov/current/title-21/chapter-I/subchapter-H/part-820.

[48] (2022). ISO/IEC 27001:2022 Information Security, Cybersecurity and Privacy Protection — Information Security Management Systems — Requirements. https://www.iso.org/standard/27001.

[49] (2023). ISO/IEC 42001:2023 Information Technology — Artificial Intelligence — Management System. https://www.iso.org/standard/42001.

[50] (2025). Guidelines on Security against Electronic Intrusion for Digital Medical Devices | MFDS Notice no2025-30. https://www.mfds.go.kr.

[51] Ludvigsen KR (2023). The role of cybersecurity in medical devices regulation: future considerations and solutions. Law Tech Hum.

[52] (2022). BSI TR-03161: anforderungen an anwendungen im gesundheitswesen, version 20. Bundesamt für Sicherheit in der Informationstechnik.

[53] Klonoff DC, Han J (2019). The first recall of a diabetes device because of cybersecurity risks. J Diabetes Sci Technol.

[54] Menon V (2026). Cybersecurity breaches in medical devices: analyzing FDA safety communications in response to patient security concerns. Front Digit Health.

[55] (2025). European action plan on the cybersecurity of hospitals and healthcare providers communication from the commission. European Commission.

[56] US Food and Drug Administration (2024). Marketing submission recommendations for a predetermined change control plan for artificial intelligence-enabled device software functions: guidance for industry and FDA staff. https://www.fda.gov/media/166704/download.

[57] Mammography quality standards act (MQSA) and MQSA program. US Food and Drug Administration.

[58] (2018). Risk Management — Guidelines.

