# Supplementary material for: Regulatory Approaches to Cybersecurity Risk Management for AI-Enabled Medical Device Software in Korea, the United States, and the European Union: Comparative Document Analysis
Source: JMIR Med Inform. 2026 Jul 30;14:e94846. doi: 10.2196/94846 (PMC13422586; doi:10.2196/94846)
Supplement: Checklist 1 [file medinform-v14-e94846-s001.docx]

**Multimedia Appendix 1**

**Standards for Reporting Qualitative Research (SRQR) Checklist**

Manuscript title: Regulatory Approaches to Cybersecurity Risk Management for AI-Enabled Medical Device Software in Korea, the United States, and the European Union: Comparative Document Analysis

Authors: Saera Jung, Kihong Son

Manuscript ID: JMIR Medical Informatics #94846

**Reference:** O’Brien BC, Harris IB, Beckman TJ, Reed DA, Cook DA. Standards for reporting qualitative research: a synthesis of recommendations. Acad Med. 2014;89(9):1245-1251. doi:10.1097/ACM.0000000000000388. PMID:24979285.

*This appendix maps each of the 21 SRQR checklist items to the corresponding section(s) of the manuscript where the item is reported.*

| **No.** | **SRQR item** | **Description** | **Manuscript location and content** |
| --- | --- | --- | --- |
| 1 | **Title** | Concise description of the nature and topic of the study identifying the study as qualitative or indicating the approach (eg, ethnography, grounded theory) or data collection methods (eg, interview, focus group) is recommended. | Title page; the term “comparative document analysis” in the title explicitly identifies the qualitative approach. |
| 2 | **Abstract** | Summary of key elements of the study using the abstract format of the intended publication; typically includes background, purpose, methods, results, and conclusions. | Abstract (Background, Objective, Methods, Results, Conclusions). |
| 3 | **Problem formulation** | Description and significance of the problem/phenomenon studied; review of relevant theory and empirical work; problem statement. | Introduction, paragraphs 1–6 (motivation, prior work on SaMD and cybersecurity, regulatory and standard-setting context). |
| 4 | **Purpose or research question** | Purpose of the study and specific objectives or questions. | Abstract (Objective); Introduction, final paragraph (“The aim of this study was to compare how three major regulatory systems…” with three sub-aims). |
| 5 | **Qualitative approach and research paradigm** | Qualitative approach (eg, ethnography, grounded theory, case study, phenomenology, narrative research) and guiding theory if appropriate; identifying the research paradigm (eg, postpositivist, constructivist/interpretivist) is also recommended; rationale. | Methods > Study Design (qualitative comparative document analysis informed by functional comparative legal research). |
| 6 | **Researcher characteristics and reflexivity** | Researchers’ characteristics that may influence the research, including personal attributes, qualifications/experience, relationship with participants, assumptions, and/or presuppositions; potential or actual interaction between researchers’ characteristics and the research questions, approach, methods, results, and/or transferability. | Methods > Researcher Characteristics and Reflexivity (disciplinary and professional backgrounds of both authors [SJ: MS in microbiology, regulatory and quality consulting for medical technologies; KS: PhD, medical informatics and AI in healthcare]; statement that neither author is employed by, or has consulted for, the regulatory agencies whose documents are analyzed; acknowledgment of potential practitioner-oriented framing arising from SJ’s submission experience and the more conceptual, informatics-oriented perspective contributed by KS; mitigation through independent review of each jurisdiction’s documents using the shared analytic framework, with differences reconciled through discussion). |
| 7 | **Context** | Setting/site and salient contextual factors; rationale. | Introduction (regulatory and standards landscape across Korea, US, EU); Methods > Data Sources and Selection (jurisdiction-specific corpus assembly). |
| 8 | **Sampling strategy** | How and why research participants, documents, or events were selected; criteria for deciding when no further sampling was necessary (eg, sampling saturation); rationale. | Methods > Data Sources and Selection (purposive selection of authoritative regulatory, guidance, and standards documents; explicit inclusion and exclusion criteria; final corpus of 10 documents listed in Table 1). No specialized qualitative analysis software was used; coding and consensus were maintained through structured author discussion |
| 9 | **Ethical issues pertaining to human subjects** | Documentation of approval by an appropriate ethics review board and participant consent, or explanation for lack thereof; other confidentiality and data security issues. | Methods > Ethical Considerations (publicly available documents; no human participants; ethics approval and informed consent not applicable). |
| 10 | **Data collection methods** | Types of data collected; details of data collection procedures including (as appropriate) start and stop dates of data collection and analysis, iterative process, triangulation of sources/methods, and modification of procedures in response to evolving study findings; rationale. | Methods > Data Sources and Selection (assembly of the regulatory corpus from publicly available sources; supplementation with peer-reviewed literature; cutoff date of March 2026). |
| 11 | **Data collection instruments and technologies** | Description of instruments (eg, interview guides, questionnaires) and devices (eg, audio recorders) used for data collection; if/how the instrument(s) changed over the course of the study. | Methods > Analytical Approach (common analytic framework mapping document content into three predefined domains—conceptual scope, premarket operationalization, and postmarket operationalization—applied uniformly by both reviewers across all jurisdictions; principles of functional comparative legal analysis used as the comparative instrument). |
| 12 | **Units of study** | Number and relevant characteristics of participants, documents, or events included in the study; level of participation (could be reported in Results). | Methods > Study Design (unit of analysis: regulatory/guidance/standards documents); Table 1 (10-document core corpus, with jurisdiction, issuing body, and year). |
| 13 | **Data processing** | Methods for processing data prior to and during analysis, including transcription, data entry, data management and security, verification of data integrity, data coding, and anonymization/de-identification of excerpts. | Methods > Researcher Characteristics and Reflexivity (independent review of each jurisdiction’s documents by the two authors using the shared analytic framework, with differences reconciled through discussion). All source documents are publicly available regulatory, guidance, and standards texts, so no transcription, anonymization, or de-identification was required. |
| 14 | **Data analysis** | Process by which inferences, themes, etc, were identified and developed, including the researchers involved in data analysis; usually references a specific paradigm or approach; rationale. | Methods > Analytical Approach (three-domain framework: conceptual scope, premarket operationalization, postmarket operationalization; functional comparative legal analysis; ISO 13485 QMS mapping; conceptual model development). |
| 15 | **Techniques to enhance trustworthiness** | Techniques to enhance trustworthiness and credibility of data analysis (eg, member checking, audit trail, triangulation); rationale. | Methods > Study Design and Researcher Characteristics and Reflexivity (use of a predefined three-domain analytic framework grounded in functional comparative legal analysis; independent review by two authors with reconciliation of differences through discussion); Methods > Data Sources and Selection and Discussion > Limitations (triangulation of the regulatory corpus with peer-reviewed literature on recalls, vulnerabilities, and postmarket monitoring). |
| 16 | **Synthesis and interpretation** | Main findings (eg, interpretations, inferences, and themes); might include development of a theory or model, or integration with prior research or theory. | Results (Cybersecurity Concepts and Definitions; Premarket Control of Software and AI-Enabled Devices; Postmarket Surveillance and Cyber-Incident Reporting); Discussion (Principal Results; Misalignment between ISO 14971 and cybersecurity risk management; AI-specific threats; governance fragmentation; QMS integration including ISO 13485 mapping in Table 5; conceptual model in Figure 2). |
| 17 | **Links to empirical data** | Evidence (eg, quotes, field notes, text excerpts, photographs) to substantiate analytic findings. | Results (Tables 2 and 3 with quoted/adapted definitions and key cybersecurity elements per jurisdiction; Table 4 with reportable-incident definitions and parallel cybersecurity reporting mechanisms). |
| 18 | **Integration with prior work, implications, transferability, and contribution(s) to the field** | Short summary of main findings; explanation of how findings and conclusions connect to, support, elaborate on, or challenge conclusions of earlier scholarship; discussion of scope of application/generalizability; identification of unique contribution(s) to scholarship in a discipline or field. | Discussion > Comparison With Prior Work; Discussion > Recommendations and Future Work; Conclusions. |
| 19 | **Limitations** | Trustworthiness and limitations of findings. | Discussion > Limitations (four explicitly enumerated limitations: lack of stakeholder interviews and quantitative data; rapidly evolving guidance landscape; non-binding nature of guidance; abstraction of the functional comparative approach from the broader legal-cultural context of each jurisdiction; mitigation steps reported for each, including triangulation with peer-reviewed analyses, explicit identification of draft versus final versions in Tables 1 and 3, framing of guidance as expected interpretations rather than enforcement standards, and confinement of claims to convergence and divergence in operational expectations). |
| 20 | **Conflicts of interest** | Potential sources of influence or perceived influence on study conduct and conclusions; how these were managed. | Conflicts of Interest section (“None declared”). |
| 21 | **Funding** | Sources of funding and other support; role of funders in data collection, interpretation, and reporting. | Funding Statement (ETRI internal fund 25YR1610; explicit statement that the funder had no role in study design, document selection, analysis, or the decision to submit for publication). |

***Note.*** *The SRQR is a 21-item checklist developed to improve the transparency of qualitative research reporting. The checklist items reproduced here follow O’Brien et al. (2014).*
